# Organizational peer support to enable rehabilitating surgical services in Northern Ethiopia

**DOI:** 10.1186/s13031-023-00515-y

**Published:** 2023-04-15

**Authors:** Meskerem Aleka Kebede, Andualem Beyene, Nurhusen Kedir, Bethelhem Abegaz, Rocco Friebel

**Affiliations:** 1 Global Surgery Policy Unit, London School of Economics and Political Science, London, UK; 2 Department of Surgery, Addis Ababa University, Addis Ababa, Ethiopia; 3 Alert Hospital, Addis Ababa, Ethiopia

**Keywords:** Surgical care, Conflict, Ethiopia, Rehabilitation

## Abstract

The ongoing violent conflict in Northern Ethiopia has caused displacement, death, and destruction. Health services infrastructure became one of the primary victims of the war, leaving millions unable to access essential surgical health services at a time when demand for surgical interventions is on the rise. Rehabilitating surgical services was identified as a priority by the federal government, regional health bureaus, and humanitarian organizations, forming an integral part in rebuilding communities after war. Under the auspices of the Federal Ministry of Health of Ethiopia, a hospital twinning program between providers in non-conflict and conflict affected areas was first introduced in December 2021, now including 13 active partnerships. The program builds on a previous best practice gained from the Ethiopian Hospital Alliance for Quality to strengthen local health care providers in regaining capabilities to serve local populations. Field experience of two hospital twinning projects have shown significant scope of organizational peer support at times of crisis, successfully enabling conflict-afflicted hospitals to regain the capacity necessary to re-introduce surgical services. While overcoming challenges such as lack of basic supplies including electricity and blood may be required to further increase the scope of this program in Northern Ethiopia, relative success highlights important lessons for similar approaches in areas affected by conflict, or natural disasters.

## Introduction

It has been more than two years since armed conflict broke out in Tigray, Northern Ethiopia, with fighting spreading to neighbouring regions in Amhara and Afar in June 2021, resulting in the internal displacement of 2 million people, material destruction, and significant loss of lives [1]. While the accurate death toll of the war remains contested, fighting and a lack of essential health services are considered the leading cause of mortality, with all involved armed groups being implicated in the destruction and looting of health care facilities [2].

Health services and health care facilities are one of the primary victims of the conflict [3, 4], either through destruction, or by being forced to reduce and discontinue services because of a lack of medicines and supplies. According to the World Health Organization, over 50% of the health facilities in Tigray were lost since the armed conflict started, leaving large parts of the population without access to the most essential health services and medicines [5, 6]. Recently, Ayder Hospital, Tigray region's main referral hospital in the capital Mekelle servicing a population of 9 million people closed its doors due to the lack of supplies, electricity, and fuel [7]. In December 2021, the Ethiopian Federal Ministry of Health announced the closure of 1500 health facilities in Amhara and Afar regions, which were rendered dysfunctional due to looting, vandalization or deliberate destruction. Those hospitals that have continued provision of services are doing so at a reduced capacity due to lack of basic supplies at the face of increasing demand, with the provision of health services further hampered by an increasing attrition of health care professionals, who may have either fled in search of safety, been directly involved in the war, or killed. Currently none of the health facilities in the region operate at a pre-war capacity [8].

Surgical services are at the forefront of delivering life-saving interventions to improve population health, particularly during times of armed violence that leads to drastic increases in demand for surgery. However, these services are particularly prone to disruption in conflict and post-conflict areas, since delivering safe surgery requires a highly trained workforce, a sterile environment, specialized equipment, different types of consumables, blood and safe anaesthesia, and post-operative care [9]. Recognizing the challenges associated with delivering surgical services, a novel hospital twinning initiative has been introduced in Northern Ethiopia to overcome critical constraints to allow the re-introduction of surgical services. As the country prepares for the initiation of peace talks to end the conflict, we aim to reflect on the developments of surgical care in Ethiopia and discuss the role of hospital twinning as an organizational peer support approach to enable rehabilitating surgical services, which is an integral part of rebuilding communities affected by war.

## Surgical care in Ethiopia pre-conflict

Peacetime surgical services in Ethiopia have seen significant improvements over the past decades. For instance, between the 2008 to 2016, the number of hospitals that provide caesarean deliveries has tripled from 87 to 253, resulting in improved access to care, ultimately reducing maternal mortality (− 39%) from 676 per 100,000 live births in 2011, to 412 per 100,000 live births in 2016 [10]. It also improved neonatal survival, which contributed to achieving MDG4 three years prior to the deadline. To develop specialised workforce skills such as in neurosurgery and pediatric surgery, Ethiopia has initially relied on overseas partnerships [11], though over the past decades, the country has forged itself as a regional training center for several East African professionals [12]. The Federal Ministry of Health has also been able to devise large-scale, government-driven comprehensive health reform initiatives focused on surgical care (*e.g.*, Saving Lives through Safe Surgery) to improve quality surgical delivery with demonstrable positive impacts on surgical leadership, logistics and supplies, building partnerships and advocacy for safe surgery. This effort includes working towards quality surgical data reporting and designing practical tools to improve access to surgery and patient outcomes [13].

Despite these achievements, the country's surgical services suffer from widespread logistical, workforce and financial constraints [14]. In 2008, Ethiopia had the lowest measured surgical rate in the world at 148 per 100,000 population [15], increasing to 192 per 100,000 population in 2019/2020 with notable differences between facility types [16]. Despite multiple efforts to address workforce capacity via home grown initiatives like increasing the number of Surgical Anaesthesia and Obstetric (SAO) trainees, or task-sharing and task-shifting among different levels of professionals, SAO density remains low at 1 per 100,000 population [17], only 1.44 anaesthesia provider per 100,000 population in 2019 [18] with majority (98%) being non-physician anaesthetists [19]. The available surgical workforce range significantly below the target set by the Lancet Global Commission in Global Surgery of at least 20 per 100,000 population by 2030, or the threshold of 5 per 100,000 populations set by the World Federation of Societies of Anesthesiologists.ranging significantly below the target set by the Lancet Global Commission in Global Surgery of at least 20 per 100,000 population by 2030. Workforce shortages are further exacerbated through an uneven distribution among rural and urban areas, for instance in 2013, 48% of all the country’s trained surgeons resided in Addis Ababa; the capital city [15].

For surgical services, extensive pre- and in-hospital waiting times are the norm [20]. Delayed care seeking and late presentation is often caused by long distances patients are required to travel to access health care facilities. A recent report suggested that on average patients travel 284.3 km, or 28.4 h to access services at specialized hospitals in Ethiopia [16]. Surgical patients are confronted with high rates of catastrophic expenditure due to a lack of financial risk protection, further contributing to delayed care seeking behavior [21, 22] and causing a substantial unmet need (approximately 5 million people) [20], with high levels of mortality and disability linked to conditions treatable through surgery. Considering this baseline, the situation was worsened by the COVID-19 pandemic. For example, a reduction in emergency and elective surgical volume was noted at several large referral surgical centers: Tikur Anbesa Specialized Hospital (TASH) (− 32% elective surgeries; − 19% emergency surgeries), and Ayder Comprehensive Specialized hospital (ACSH) and Tibebe Ghion Specialized Hospital (TGSH) (Combined) (− 80% elective surgeries; − 20% emergency surgeries) [23, 24].

## Impact of conflict on surgical care delivery in Ethiopia

While there is paucity of reliable information on the devastation caused by the war in Northern Ethiopia, reports from humanitarian agencies signify the severe damage to health services occurred in all three regions. Two thousand nine hundred and twenty one health facilities in Amhara and Afar regional states have reportedly sustained destruction, including 40 damaged hospitals, 450 health centres and health posts in the Amhara region alone [25]. At the lowest level of organization in the three-tiered Ethiopian health system, a total of 539 facilities capable of providing surgical care have been damaged in Amhara and Afar, resulting in substantial loss of surgical capacity within the regions. Additionally, supply-chain issues are rampant, with the health coordinator at the International Committee of the Red Cross delegation in Ethiopia reporting dwindling supplies in conflict afflicted regions. In parts of Tigray, single-use items including gloves, surgical materials and chest drains are being washed and reused, ultimately increasing the risk of infections. A lack of basic supplies also leads to common replacement of disinfectant for salt to clean wounds, further risking patient safety and hampering outcomes of surgical care [26]. It was estimated that the loss of equipment and supplies at primary and general hospitals and health centers in the Amhara region amounts to 1,512,872,815 ETB (approximately 28,658,321.94 USD) [27].

Surgical need resulting from casualties of the war, and the general population, now widely exceeds available surgical resources in Northern Ethiopia. In a series of presentations at the Ethiopian Medical Association’s annual conference, surgeons working at hospitals in proximity to the war zones provided insights on the impact of the war on surgical services. Presenters noted overwhelmed hospitals with causalities arriving every few hours, including soldiers and civilians with various gunshot wounds, and other surgical emergencies. However, many hospitals located in proximity to the war lines had to discontinue elective surgeries and cold case follow-ups due to resource and workforce shortages. Most of these hospitals were staffed with volunteer surgeons who at certain instances were being supported by local community volunteers.

The inability to serve the operative needs of the general population, including the loss of access to facilities among pregnant women, risks reversing the gains made over the past decades. For example, an increase in childbirth related injuries is likely to raise cases of obstetric fistula, one of the most debilitating complications of traumatic childbirth with long term consequences for the woman, and the wider community, when untreated. An uneven distribution of demand pressures also carries spill-over impacts on surgical services beyond hospitals in the vicinity of the war zone. Ripple effects have caused significant increases in demand for surgical services in other parts of the country [28], leading to backlogs and undermining health services resilience with adverse impacts on access for local populations.

## Rehabilitating surgical services in Ethiopia

There is an urgent need to rehabilitate health care service in Ethiopia. The Federal Ministry of Health estimates that close to 90 million USD will be required for the first phase of a planned recovery program, which comprises of a targeted rehabilitation of minor surgery care, trauma care, and essential health services. An additional estimated 187 million USD will be required for the second and third phases of implementation, which comprises the resumption of major surgical services and intensive care unit functionalities among several other planned activities [25]. This budget is planned to be mobilized from government financing, private sector, donor agencies and Ethiopian diaspora.

The Federal Ministry of Health of Ethiopia, in partnership with regional health bureaus, and humanitarian organizations, has been organising rehabilitation of services in government-controlled areas. Data on the extent of damage was collected, a rehabilitation plan devised, resources mobilized, and relief efforts coordinated. This included the design of a phased approach to allow hospitals to gradually return to pre-conflict service delivery capacity, involving steps such as medical supplies delivery and professional support.

An integral part of the rehabilitation process forms the hospital twinning initiative, first initiated in mid-December 2021 once federal government regained territorial control, between Federal and Addis Ababa Regional Health Bureau (ARHB) hospitals located in Addis Ababa and conflict affected hospitals in Amhara Regional State [25, 29]. Hospital twinning acts as an institutional peer support mechanism that encompasses all forms of health care, to enable quick scale-up of service delivery through organizational support with human resources, financial assistance, equipment, medical supplies, and psychosocial support. While participation in the program is voluntary, institutions are selected based on set of criteria including human resource availability, level of expertise, area of specialty and financial resource availability. Currently, there are 13 active twinning partnerships between federal hospital and recipient hospitals in Northern Ethiopia and 17 in the process of being established (see Table 1). Additional partnerships are being initiated in other parts of the country to support institutions affected by conflict in Oromia and Benishangul Regional States. While primary focus of the initiative is hospital rehabilitation, regional health bureaus also participate to support health care centres in solidarity.

**Table 1:** Overview of hospital twinning partnerships in Northern Ethiopia

No.	Twin Hospital (location)	Supporting Hospital (location)
*Hospitals in active implementation of the twinning program*		
1	Dessie Comprehensive Specialized Hospital (NW)	St Pauls Millennium Medical College (AA)
2	Boru Meda General hospital and Kelewan Health Center (SW)	St Peters Specialized Hospital (AA)
3	Kombolcha General Hospital (SW)	Alert Hospital (AA)
4	Haik Primary Hospitals (SW)	Eka Kotebe Hospital (AA)
5	Mehal Meda General Hospital (NS)	Menilik II Referral Hospital (AA)
6	Debre Sina Primary Hospital (NS)	Ras Desta General Hospital (AA)
7	Wereylu Primary Hospital (NS)	Yekatit 12 Hospital (AA)
8	Bati Primary Hospital (NW)	AaBet Hospital (AA)
9	Jamma Dogolo Primary Hospital (SW)	Zewditu Primary Hospital (AA)
10	Kemisse General Hospital (OSZ)	Tirunesh Bejing Hospital (AA)
11	Molale Primary Hospital (NS)	Ghandi Memorial Hospital (AA)
12	Woldia Comprehensive Specialized Hospital (NW)	Tikur Anbesa Specialized Hospital (AA)
13	Ataye Primary hospital (NS)	Werabe Comprehensive Specialized Hospital (SNNPRS)
*Hospitals with an initial assessment but no other activities*		
1	Delanta Hospital (SW)	Jimma University Medical Center (ORS)
2	Mersa Primary Hospital (NW)	Hawassa University Hospital (SidRS)
3	Wadla Primary Hospital (NW)	Wolayita Sodo University Hospital (SNNPRS)
*Hospitals that are undertaking preparatory work to take part in the twinning program*		
1	Akesta Hospital (SW)	Hiwot Fana Specialized Hospital (HRS)
2	Tefera Hailu Specialized Hospital (WHZ)	Dilla University Hospital (SNNPRS)
3	Ade Arkay Primary Hospital (SGZ)	Jijiga Specialized Hospital (SomRS)
4	Zikuala Primary Hospital (WHZ)	Nigist Eleni Mohammed Memorial Teaching Hospital (SNNPRS)
5	Tenta Primary Hospital	Mizan Tepi Hospital (SNNPRS)
6	Lalibela General Hospital (SW)	Arba Minch University Hospital (SNNPRS)
7	Amdework Primary Hospital (WHZ)	Welkite Hospital (SNNPRS)
8	Shewa Robit Primary Hospital (NS)	Mede Welabu Hospital/Adama Medical Center (ORS)

*NW* North Wollo Zone; *SW* South Wollo Zone; *NS* North Shewa Zone; *OSZ* Oromia Special Zone; *WHZ* Wag Himra Zone; *SGZ* South Gonder Zone; *AA* Addis Ababa; *SNNPRS* Southern Nations and Nationalities Regional State, *ORS* Oromia Regional State; *SidRS* Sidama Regional State; *SomRS* Somali Regional State. Note: this table was produced based on a Federal Ministry of Health report January 2022

The Federal Ministry of Health has pursued hospital twinning as an approach to rehabilitate surgical capacity for several reasons. The twinning initiative was based on a previously successful and continuing model called the Ethiopian Hospitals Alliance for Quality (EHAQ); a hospital collaborative learning platform launched in 2012 for the improvement of quality of care that is currently in its 4^th^ implementation cycle. Each cycle focused on a selected health care service theme and connected hospitals across the country as clusters to enable resource sharing and peer support. Evaluations of EHAQ have shown that the collaboration played an instrumental role in promoting clean and safe hospitals, improving patient satisfaction and maternal and child health services [30, 31]. The program also offers a sustainable, locally driven approach that reduces the reliance on foreign inputs, which provides an opportunity to overcome limitations of commonly applied approaches in rehabilitating surgical services, where the primary responsibility of care delivery and rehabilitation is through humanitarian agencies. Particularly, it helps address a lack of clear understanding of local needs and norms, and ensures accountability.

Despite the opportunities inherent to the twinning program, there remain significant challenges to the chosen approach in Ethiopia. The primary challenge pertains resource constraints within the non-conflict affected hospitals. Some of the participating providers experienced significant rises in demand pressures due to the treatment of war casualties referred from the frontlines, and a further reduction of an already stretched health care workforce by commissioning staff to attend conflict-afflicted hospitals [28]. A partnership which is resource intensive could therefore further strain routine services, negatively impacting access and outcomes of patients in non-conflict areas. This is also likely to compromise the mental and physical wellbeing of the workforce when having to cope with an unprecedented burden of health care needs. Second, even though surgical services have been re-established, fear of stigma (particularly in populations that experienced gender-based violence), and a lack of awareness may hinder health seeking behavior within the communities the hospitals serve. Building trust and awareness is therefore necessary to address the needs of local populations, beyond the infrastructure needs. Finally, while hospital twinning may provide a quick solution to restart surgical services, the sustainability of this approach beyond the immediate conflict period remains unknown. Even though one of the founding principles of the twining program is sustainability, continuous motivation, and commitment from supporting hospitals could be challenged in the medium to long-term, particularly when negatively affecting the service provision at the non-conflict affected hospital. Lack of motivation and long-term engagement may leave the paired hospitals vulnerable to losing their mentorship and support before they are fully rehabilitated. To address this concern, some hospitals have already devised creative solutions, such as making paired hospitals training sites for their residents to ensure continued partnership.

Pending a full evaluation report of the twinning program, we present the field experience of two examples and highlight their achievements and challenges faced during the implementation. Tikur Anbesa Specialized Hospital (TASH) and Alert Hospital (ALERT) were one of the first providers to join a partnership with a conflict-affected hospital in Northern Ethiopia (Woldia Comprehensive Specialized Hospital and Kombolcha General Hospital, respectively), allowing for a detailed understanding on how the model works and its intended objectives in addressing infrastructural damage, and the loss of workforce and other necessary resources.

## Case study 1: Tikur Anbesa Specialized Hospital–Woldia Comprehensive Specialized Hospital

Tikur Anbesa Specialized Hospital (TASH) is a hospital affiliated to Addis Ababa University and one of the pioneer hospitals to participate in the twining program. The hospital started implementing their partnership with Woldia Comprehensive Specialized Hospital (WCSH) in December 2021, as soon as the Ethiopian government regained control of Woldia town the administrative capital of Northern Wollo Zone. TASH comprises of one of the most experienced surgical team in the country, with expertise in deploying surgical professionals to provide emergency surgical care during several small-scale conflicts in Somali region, Benishangul, and others. WCSH serves an estimated 3 million people from Amhara and the neighboring Tigray and Afar regions. The hospital has 17 consultants, 45 general practitioners, 172 nurses, and 66 midwives [32].

After an initial familiarization with the team at WCSH to define the scope of the partnership, including the expected allocation of roles and responsibilities, the primary task of the TASH team was to conduct a rapid assessment of the damages and the resources required to rehabilitate care at WCSH. Situation reports revealed that the hospital had ceased all services for a period of two weeks when Woldia was captured, though local faith leaders then organized health professionals to enable the hospital to continue limited emergency services. Approximately 30 health professionals supported by volunteers were working at the WCSH until government forces re-captured the city on the 6^th^ of December 2021. It was found that no health professional had died from WCSH, with many returning quickly to resume their services. However, unlike human resources, the assessment also revealed that most of the operating room equipment (*e.g.*, anesthesia machine, operating room lights, monitors), operating tables, and most drugs were unavailable, damaged, or looted.

Following this rapid assessment, a team of professionals with various expertise (including 5 consultant surgeons, surgical residents, anesthetists, biomedical engineers) arrived in Woldia with the necessary equipment, and supplies amounting to around 8 million ETB (~ 154,000USD). In addition to external support, professionals from TASH were able to maintain equipment with reparable damage and set-up two functioning operating rooms to provide surgical services. Emergency surgeries like caesarean section, laparotomy for acute abdomen and orthopedic trauma surgeries were restarted within two weeks post conflict.

To further strengthen this partnership, both organizations established WCHS as a training site for surgical and obstetric trainees from Addis Ababa University. To ensure sustainable support and sufficient workforce capacity as WCSH strives to return to pre-war capacity. Addis Ababa University continues to provide operative services with mostly non-physician anesthetists providing anesthetic care. However, further quality training programs for other health care professionals, including anesthesia and operating room nurses, will be required to strengthen local surgical capacity sustainably.

Despite the substantial progress made through the twinning with TASH, WCSH experiences continuous infrastructural challenges that hamper the rehabilitation efforts. A lack of electricity and fuel to power generators periodically obstructs the surgical teams to perform procedures, reducing the overall impact of the rehabilitation efforts. Additionally, shortages of essential medications including blood often causes referrals to nearby hospital if expertise is available. TASH is in the process of engaging other partners to relive the financial and resource constraints faced by WCSH.

## Case study 2: Alert Hospital–Kombolcha General Hospital

Alert Hospital was paired with Kombolcha General Hospital (KGH), located in the industrial Kombolcha town. It was a newly established 200 bed hospital, inaugurated in January 2021. KGH had been operational only for about 5 months before being engulfed in the advancing conflict. Alert hospital was selected based on its willingness to support the rehabilitation effort, as well as the proximity of Kombolcha town approximately 390 Kms from Addis Ababa. The Alert team arrived in Kombolcha to initiate the partnership on the 9th of December 2021. The initial team comprised of 6 management team members, with their primary task to perform a situation assessment and map available resources. Reports indicated severe damage to hospital infrastructure, the hospital reported it has sustained a loss valued approximately 800 million ETB (~ 15.1 million USD) additionally, the Alert senior management team was also shifted to Komolcha to closely oversee the implementation of rehabilitation efforts.

To restart health care services, an initial focus was given to initiating basic emergency medical and surgical care. Following the initial assessment, the team recognized that much of the 164 team members (98 out of 164 are clinical team members while the others are supports staff) of KGH had returned to work. Yet, this only covered approximately 50% of the hospitals staffing needs, which prompted Alert hospital to send an additional 7 team members comprising of one general surgeon, five operating room nurses and one laboratory technologist, to support KGH. Significant rehabilitative gains were made early in the partnership. For example, within two weeks, one operating room with two operating tables became functional along with a 12-bed surgical ward and health care professionals were able to perform 38 cesarian deliveries. Equipment and essential medicines were purchased or repaired to address the identified need, which was afforded through donations of 15 million Ethiopian Birr (approximately 290,000 USD), mobilized from Alert hospital and the wider hospital community as well as in kind donations.

The key challenge faced by Alert hospital in its rehabilitation efforts related to the nature of a newly inaugurated hospital. When the conflict affected KGH, the administration had just begun with staff recruitment, and therefore any rehabilitation efforts could not build on existing human resources capacity. To address this challenge, local volunteers including military personnel, were mobilized to provide support during the initial period of the twinning program, with recruitment efforts intensified to scale up the local health care workforce.

## Lessons for rehabilitating surgical services in conflict-inflicted areas

Despite substantial resource constraints, the Ethiopian health care system has made significant progress in providing access to high-quality health services for its population over the past decades. However, the ongoing conflict in Northern Ethiopia threatens to undo historic gains, putting millions of people at risk of death and morbidity due to a lack of access to the most basic health care interventions. Rehabilitating surgical services has therefore been a priority in Ethiopia, with the hospital twinning program offering a low-cost, local-driven approach to rehabilitate hospital services in areas affected by war. While there has not been a formal evaluation of this program yet, field experience has shown substantial short-term advantages over traditional humanitarian relief, offering valuable lessons to other conflict-inflicted areas, globally.

The twinning program is an example of how health care systems can build on existing expertise and resources, particularly when conflict or natural disasters are affecting only parts of a country. It demonstrates the importance of local government ownership coupled with the principle of solidarity in sharing inputs between providers. While the redistribution and mobilisation of resources strongly depends on information of incurred damages, it highlights the need for assessment tools to accelerate the recovery process. This could be done through employing technologies such as geospatial mapping of health care facilities, previously used to assess access to care in Yemen [33]. However, the twinning program in Ethiopia has also highlighted significant challenges, particularly regarding resources sharing in an environment that is characterised by widespread scarcity. To support the long-term sustainability, it may therefore be beneficial to explore the use of tele educative interventions to upscale local-level expertise without diverting workforce capacity from other parts of the country. Importantly, any interventions should be oriented towards compressive surgical care system strengthening and aimed at providing wholistic peri-operative surgical and anesthesia care.

The primary factor in ensuring sustainable strengthening of health care systems across fragile areas remains peaceful resolution of the conflict. The Ethiopian experiences demonstrate the value of leveraging local resources and innovative models to ensuring the quick rehabilitation of services in conflict affected areas.

## Data Availability

Not applicable.

## Abbreviations

EHAQ: Ethiopian Hospitals Alliance for Quality
TASH: Tikur Anbesa Specialized Hospital
WCSH: Woldiya Comprehensive Specialized Hospital
KGH: Kombolcha General Hospital
